# The Association between Embryo Development and Chromosomal Results from PGT-A in Women of Advanced Age: A Prospective Cohort Study

**DOI:** 10.3390/jcm13020626

**Published:** 2024-01-22

**Authors:** Pornchanit Santamonkunrot, Sonsiri Samutchinda, Pornsri Niransuk, Chonthicha Satirapod, Matchuporn Sukprasert

**Affiliations:** Reproductive Endocrinology and Infertility Unit, Department of Obstetrics and Gynecology, Ramathibodi Hospital, Mahidol University, Bangkok 10400, Thailand; spornchanit@gmail.com (P.S.); bright-sonsiri@hotmail.com (S.S.); tik.punnpunn@gmail.com (P.N.); chonthicha_lek@yahoo.com (C.S.)

**Keywords:** preimplantation genetic testing, aneuploidy, advanced-aged women, morphology, morphogenetic, embryo development

## Abstract

Embryo morphology and morphokinetics have been studied for their association with euploid embryos. However, the results are controversial, especially in the advanced-aged women group, when the risk of aneuploidy increases significantly. This prospective cohort study evaluated the association between embryo development between day-3 cleavage and day-5 blastocyst stages and euploidy rates, determined using preimplantation genetic testing for aneuploidy (PGT-A). Embryos from women aged 35 years and above who underwent intracytoplasmic sperm injections and PGT-A were studied. Day-3 cleavage-stage embryos were evaluated for their cell number, and day-5 blastocyst-stage embryos were evaluated for their morphological grade. Embryo development from day 3 to day 5 was categorized as either good or poor development and evaluated for its association with the PGT-A results. We evaluated 325 embryos from 101 infertile couples. It was found that 55.17% of blastocysts with good development and 29.83% with poor development were euploid. A significant association was found between embryo development and euploidy rates in advanced-aged women (*p* < 0.001). Also, there were significantly higher rates of euploid embryos with good blastocyst morphological grades, especially blastocyst expansion grades and trophectoderm grades. In conclusion, embryo morphokinetics shows promising results in predicting euploidy in advanced female age.

## 1. Introduction

A significant contributor to assisted reproductive technology (ART) failures and increased miscarriage rates is chromosome aneuploidy, which is particularly prevalent in advanced-aged women. Recently, the widespread adoption of preimplantation genetic testing for aneuploidy (PGT-A) has emerged as a promising indicator of ART success [1]. Nonetheless, PGT-A has certain limitations and associated risks. It demands increased resources and labor from embryologists because not all embryos survive the biopsy. In addition, it extends the time required to achieve pregnancy and incurs higher costs. The overall cost-effectiveness and improvement in cumulative live birth rates of PGT-A are still in question [2]. A recent meta-analysis concluded that PGT-A increases live birth rates and ongoing pregnancy rates [3]. However, another meta-analysis has reported that PGT-A did not improve live birth rates in the general population, and outcomes were only improved in women aged over 35 years old [4]. Thus, there is a need for a more cost- and time-effective method.

Morphokinetics is an essential process of cell division during embryo development. During blastulation, cell division involves the intricate process of kinetochore attachments to microtubules, which requires cohesion molecules for precise chromosome separation and specialized proteins for accurate gene expression. Errors in detection and DNA repair mechanisms can give rise to aneuploid embryos; therefore, deviations from the normal blastulation process may predict aneuploidy. Delayed morphokinetics has been suggested as a potential indicator of aneuploid embryos because it may reflect the detection of errors during embryo development, resulting in slower cell division. Conversely, rapid growth with short cell cycles may not provide sufficient time for complete DNA replication and repair before chromosomal alignment, thereby increasing the risk of aneuploidy [5]. A recent systematic review and meta-analysis identified specific parameters that show some promise for predicting ploidy status [6], t8 and t9, which denote the time elapsed from insemination to the completion of the 8-cell and 9-cell divisions, respectively, and tB and tEB, which represent the durations from insemination to the formation of a full blastocyst and an expanded blastocyst, respectively. These parameters indicate the time it takes for embryos to reach the blastocyst stage and, potentially, their ploidy status. However, despite these findings, time-lapse microscopy has not gained widespread adoption, because of concerns about its cost-effectiveness and uncertainty regarding its overall benefits and necessity.

Morphological grading is a widely adopted, non-invasive method for selecting embryos for transfer. An observational study noted that the morphology of blastocysts served as a predictive factor for their chromosomal status [7]: blastocysts exhibiting excellent morphology exhibited the highest rates of euploidy, while those with poor morphology exhibited the lowest rates. These findings align with those of numerous prior studies that have consistently reported an association between blastocyst morphology and euploidy rates [8,9,10,11]. However, a retrospective analysis observed that this association was unclear among women over the age of 35 [12].

Previous studies have shown that studying day-3 to day-5 embryo development can predict improved ongoing pregnancy rates when development is optimal [13]. Notably, Magli et al. reported a correlation between the number of cells present during the day-3 cleavage stage and chromosomal abnormalities, with the lowest occurrence of abnormalities observed in embryos with 7–8 cells [14]. However, whether morphology or morphokinetics predicts euploidy rates is controversial and requires further research, especially in advanced-aged women. Therefore, this study aims to clarify the association of embryo development between the day-3 cleavage stage and the day-5 blastocyst stage with euploidy rates using PGT-A, a surrogate outcome of ART, in advanced-aged women.

## 2. Materials and Methods

### 2.1. Study Design and Population

This was a prospective cohort study. The procedures and protocols for embryo analysis were ethically approved by the Institutional Review Board (MURA2022/764) of the Faculty of Medicine Ramathibodi Hospital, Mahidol University. The data collection period spanned from December 2022 to August 2023. All patients provided their informed consent for data use and willingly agreed to participate in this study. The inclusion criteria for this study were embryos from women aged 35 years to 46 years old who were undergoing both intracytoplasmic sperm injection (ICSI) and PGT-A of the blastocyst. All controlled ovarian stimulations were performed using a gonadotropin-releasing hormone (GnRH) antagonist protocol. Embryos from couples with severe male factors (severe oligozoospermia and azoospermia) or that did not develop into blastocysts on day 5 of development were excluded from this study.

### 2.2. Embryo Assessment

Transvaginal oocyte retrieval was conducted after a standard GnRH antagonist ovarian stimulation protocol followed by ICSI. The embryos were cultured individually until the fifth development day (106–108 h after ICSI). Fertilization status was evaluated between 16 and 18 h following ICSI, whereby confirmation was based on the presence of two pronuclei and two polar bodies. Approximately 66–68 h after ICSI, cleavage-stage embryos were assessed per the Istanbul Consensus [15] and the number of cells evaluated. At this time, cleavage-stage embryos should be in the 8-cell stage. According to the Istanbul Consensus, an optimal day-3 embryo would typically have eight equally sized mononucleated blastomeres with less than 10% fragmentation. Therefore, we categorized day-3 cleavage-stage embryos into two groups: good quality (with 7 or 8 cells) or poor quality (with fewer than 7 or more than 8 cells). After 106–108 h following ICSI, the blastocysts were graded, biopsied, and vitrified. The blastocyst grading was performed by a senior embryologist using the Gardner and Schoolgraft embryo grading system [16]. The grading of blastocysts involves the evaluation of three key aspects: the rate of cell expansion, inner cell mass (ICM), and trophectoderm (TE) grade [17].

The blastocyst expansion grade was characterized using the following numerical scale:

- Grade 1 (early blastocyst): the blastocoel occupies less than half the volume of the embryo;
- Grade 2 (blastocyst): the blastocoel occupies greater than or equal to half the volume of the embryo;
- Grade 3 (full blastocyst): the blastocoel completely fills the embryo;
- Grade 4 (expanded blastocyst): the blastocoel volume surpasses that of a full blastocyst, characterized by a thinning of the zona pellucida;
- Grade 5 (hatching blastocyst): the TE begins to herniate through the zona pellucida;
- Grade 6 (hatched blastocyst): the blastocyst has completely escaped from the zona pellucida.

ICM grade was assessed based on the following three grades:

- Grade A: a tightly packed ICM with numerous cells;
- Grade B: a loosely grouped ICM with several cells;
- Grade C: very few cells are disorganized.

The TE grade was also categorized into three grades, as follows:

- Grade A: TE with many cells forming a cohesive epithelium;
- Grade B: few cells form a loose epithelium;
- Grade C: very few large cells.

The blastocyst morphological grade was organized into three distinct groups, good, moderate, and poor, as illustrated in Table 1. Within these groups, the development of embryos was categorized into two subgroups: good development and poor development groups. Good development is defined as embryos originating from good-quality day-3 embryos that developed either good or moderate blastocyst morphological grades. The rest were considered to be in the poor development group.

### 2.3. PGT-A

On the fifth day of development (106–108 h after ICSI), the embryos were removed from the incubator for evaluation and biopsy. Approximately 3–5 TE cells were gently aspirated using a biopsy pipette. The cells were then carefully washed in phosphate-buffered saline (PBS) and placed in microtubes containing 2 μL of PBS. The isolated cells were then amplified and analyzed using next-generation sequencing (NGS). The results were reported in terms of chromosomal status, whereby embryos were categorized as euploid, mosaic, or aneuploid, and notably, “complex aneuploidy” was reserved for embryos exhibiting aneuploidies in two or more chromosomes.

### 2.4. Outcome Measures

The primary outcome of this study was the association between embryo development from day-3 cleavage to day-5 blastocyst stages and euploidy rates in advanced-aged women, where euploidy rates were compared using developmental status. The secondary outcome was an evaluation of the association between blastocyst morphological grade and euploidy and aneuploidy rates within age groups.

### 2.5. Sample Size Calculation and Statistical Analysis

There are currently no published studies on the development of blastocysts using a morphological assessment of the cleavage to blastocyst stage determined by PGT-A. We established the significance level (Type I error; 0.05) and power (Type II error; 0.2) of this study on the basis of a comparison of two independent proportions. A preliminary pilot study was conducted using data obtained from our center. Among the subsets of euploid embryos, 26% exhibited good development and 11% displayed poor development. The observed difference in developmental rates between these two groups was 15%, and the ratio between poorly developed embryos and embryos with good development was 1:2. The calculated sample size required for this study was 270 embryos. Of these, 180 embryos had good development, and 90 embryos had poor development. Continuous variables are presented as means and their corresponding standard deviations if the data exhibited a normal distribution. The median was the representative value for variables that did not follow a normal distribution. Chi-squared tests were employed to identify statistically significant differences among the categorical variables. Multivariate logistic analysis was performed to assess the relationship between embryo development and euploidy rates derived from the PGT-A results. A significance level of *p* < 0.05 was considered statistically significant for all analyses. All statistical analyses were performed using STATA, version 18.0.

## 3. Results

In this study, 325 blastocysts were subjected to biopsies for PGT-A. Table 2 shows the baseline characteristics of the 101 infertile couples included in this study. The mean female age was 39.19 ± 2.61 years with the oldest female aged 45 years and 8 months, and of the female participants, 36.63% were aged between 35 and 37 years (Group 1), 23.76% were aged between 38 and 40 years (Group 2), and 39.60% were aged ≥40 years (Group 3). The median number of retrieved oocytes was eight per patient. The metaphase II (MII) oocyte rate was 80.58 ± 16.52%. The fertilization and cleavage rates were 80.58 ± 16.52% and 100%, respectively. Furthermore, the median number of total day-3 cleavage and day-5 blastocyst embryos was five and three per woman, respectively. As female age increased, there was a significant decrease in the number of retrieved oocytes, cleavage, and blastocyst embryos. Of the embryos, 127 (39.08%) were from group 1, 95 embryos (29.23%) were from group 2, and 103 embryos (31.69%) were from group 3, as shown in Table 3.

Most embryos exhibited good cleavage (7–8 cells) on day 3: 81.23% good quality and 18.77% poor quality (Table 3). However, the distribution was not significantly different among the various female age groups (*p* = 0.532). In addition, the blastulation rate, 71.02 ± 22.16%, was not statistically significantly different between the age groups (*p* = 0.785). Most blastocyst grades were categorized as poor quality (65.54%), followed by moderate quality (32.00%) and good quality (2.46%).

From the day-3 cleavage stage to the day-5 blastocyst stage, most embryos displayed poor development (73.23% vs. 26.77%). The three female age groups exhibited 31.50%, 32.63%, and 15.53% good embryo development, respectively; however, the older age groups exhibited statistically significant (*p* = 0.008) decreased rates of good embryo development.

According to the PGT-A results, the overall euploid rate was 36.62% (n = 119). Among the female age groups, the euploid rate was 52.76% (n = 67) for group 1, 34.74% (n = 33) for group 2, and 18.45% (n = 19) for group 3. The number of good blastocyst morphological grades varied among the age groups: 4.72% for Group 1, 0% for Group 2, and 1.94% for Group 3; moderate-quality: 33.07% for Group 1, 40.00% for Group 2, and 23.30% for Group 3 (*p* = 0.018). The types of aneuploidies are detailed in Supplementary Table S1, with the most common type being complex aneuploidy, accounting for 58.33% of cases.

As detailed in Table 4, 55.17% of embryos with good development were euploid, and 29.83% of embryos with poor development were euploid (*p* =< 0.001). Subgroup analysis was performed by female age group, detailed in Supplementary Table S2. Good-development embryos have a higher chance of being euploid in all age groups: Group 1 67.50% vs. 45.98%, *p* = 0.024; Group 2 48.39% vs. 28.12%, *p* = 0.052; and Group 3 37.50% vs. 14.94%, *p* = 0.072.

In addition, the factors associated with euploidy rates were female age, blastocyst expansion grade, TE grade, blastocyst grade, and embryo development, as determined using univariate logistic analysis (Table 5). Women in the age groups of ≥35 to <38 years and ≥38 to <40 years had significantly higher rates of euploid embryos compared to women aged ≥40 years. The relative risk (RR) was 2.86 (95% CI 1.85, 4.43, *p* < 0.001) for the first group and 1.88 (95% CI 1.15, 3.08, *p* = 0.11) for the second group. Embryos with a blastocyst expansion grade ≥4 had a significantly higher chance of being euploid compared to those with a grade <4. The RR was 2.06 (95% CI 1.55, 2.74, *p* < 0.001) for embryos with good expansion. Embryos with an ICM and TE grade A had a 100% euploidy rate. Although ICM grade B tended to have a higher chance of being euploid than grade C, this difference was not statistically significant (RR 1.43, 95% CI 0.70, 2.91, *p* = 0.320). Embryos with grade B TE had a significantly higher chance of being euploid compared to grade C (RR 1.82, 95% CI 1.12, 2.98, *p* = 0.017). Good embryo development had a significantly higher chance of being euploid (RR 1.85, 95% CI 1.41, 2.43, *p* =< 0.001). Multivariable analysis was performed with female age, blastocyst expansion score, and embryo development, detailed in Supplementary Table S3. Female age (age ≥ 35 to <38 RR 2.48; 95% CI 1.61, 3.83, age ≥ 38 to <40 RR 1.67; 95% CI 1.03, 2.70, *p* < 0.001) and blastocyst expansion score (score ≥ 4 RR 1.79, 95% CI 1.24–2.58, *p* = 0.002) were found to be the most important contributing factors that predict euploidy rates.

## 4. Discussion

This study evaluated the association between embryo development and chromosomal results using PGT-A, with a focus on advanced-aged women. Our analysis revealed a significant association between embryo development between the day-3 cleavage stage and the day-5 blastocyst stage and euploidy rates. Also, embryos with good blastocyst morphological grades were more likely to be euploid. These data indicate that the development and morphological characteristics of blastocysts, such as their expansion, ICM, and TE grade, can provide valuable insights into their chromosomal status.

Overall, the rate of MII oocytes, fertilization rate, cleavage rate, blastocyst development rate, and successful biopsy were all above the laboratory competency value stated in The Vienna Consensus 2017 from the European Society of Human Reproduction and Embryology (ESHRE) Special Interest Group of Embryology and Alpha Scientists in Reproductive Medicine [18]. In addition, no failure of DNA amplification occurred, and the mosaicism rate was <5%. Euploid rates decreased as age increased. In addition, an increasing rate of complex aneuploidy was found with increasing age. These results are consistent with a study conducted by Franasiak et al. [19] that analyzed data from 15,169 TE biopsies and with recent data reporting that the risk of aneuploidy is increased by approximately 10% per year of increased female age [20].

Good embryo development from the day-3 cleavage stage to day-5 blastocyst stage was found to be associated with euploid status of the embryos as it defines a good cell division process. The most important indicator of embryo viability is cell division [15]. Existing research has consistently demonstrated that a too-slow or too-fast cleavage rate harms the implantation rate [21,22,23,24]. Notably, although frequently considered, fragmentation in the cleavage stage has not been correlated with aneuploidy in previous investigations, only correlated with mosaicism [15,25]; hence, it was not included as an evaluation parameter in this study. Genome activation typically occurs during the 4-8 cell stage [26]. Key developmental processes occur after genome activation, differentiation to form ICMs, and TE to develop blastocysts, which could be disturbed by genetic abnormalities. This study initiates the evaluation of morphological and developmental factors of embryos post-genome activation, specifically commencing assessment after the cleavage stage to identify chromosomal abnormalities. A delay in development might result from irregular activation of the spindle assembly checkpoint, while aneuploid embryos encounter challenges in aligning with the metaphase plate [27]. Conversely, an excessively rapid development can result in disorderly or abnormal divisions [28]. Recent time-lapse microscopy studies have found that kinetic markers of too-slow and too-fast blastocyst formation were related to aneuploid embryos [29,30,31]. This study was designed similar to using time-lapse microscopy to predict euploidy but suggests a more cost-effective and simple method.

The Gardner and Schoolcraft grading system consists of three components: blastocoel expansion, quality of the ICM, and TE grade. However, previous studies have yielded conflicting results regarding which parameters best predict euploidy and implantation rate. Recent studies have reported that blastocoele expansion is the most critical predictor of live birth [32]. Several studies also found that TE quality was independently associated with blastocyst ploidy and/or live birth rates [12,33,34,35,36]. Some studies have also reported a similar association with ICM grade [5,11,12,37]. A retrospective study found that blastocyst morphological grade is associated with aneuploidy, and TE grade has a greater predictive power than ICM grade [9]. Other retrospective studies have found that all three parameters are relevant to euploidy rates [10,38]. The current study also found that blastocyst expansion and TE grades were associated with euploidy rates in advanced-aged women, probably because the TE cells were biopsied for PGT-A. The quality of TE cells represents good cell division and the possibility of euploidy. Most prior studies did not subgroup patients into age groups and did not emphasize data in advanced age groups. Only one retrospective study performed a subgroup analysis [12]. It has been reported that in females aged <35 years, blastocyst morphology was associated with euploidy rates; however, in females aged ≥35 years, this association was lost, and good morphology did not have the highest euploid rates. The current study, by focusing on advanced-aged women, provides new data that blastocyst expansion grade and TE grade are associated with euploidy rates in this population.

From the univariate logistic regression analysis, factors found to have a significant association with euploidy rates were female age, blastocyst expansion grade, TE grade, blastocyst morphological grade, and embryo development. In the multivariate logistic regression analysis, only female age and blastocyst expansion grade were significant factors. The reason may be that in the advanced-age population, there are fewer embryos with good development but with increased aneuploid rates. The number of embryos with good development that are euploid might be so small that other factors (increasing female age and blastocyst expansion grade) overcame its significance. Future research with larger sample sizes will provide more data on this factor.

This study contains several unique aspects that are invaluable to the medical world. First, it focused on advanced-aged women and conducted analyses within different age groups, a methodology not commonly employed in previous research. Second, all PGT-A cycles were exclusively performed at a single specialized reproductive center, thereby enhancing the reliability and consistency of this study’s findings. Moreover, all PGT cycles utilized NGS, a cutting-edge technology that adds to the robustness of the results. Third, this study included embryos of varying quality for biopsy, allowing for a more comprehensive assessment of euploidy rates. In contrast, many prior investigations exclusively biopsied good-quality blastocysts. Fourth, a single, highly experienced embryologist undertook the embryo grading process. Given the subjective nature of embryo evaluation, this approach promotes greater standardization of the grading process. Lastly, this study adopted a prospective cohort design, which provided a controlled and structured approach to data collection, in contrast to the retrospective nature of many earlier studies.

However, the current study has several limitations. First, the primary outcome focused on euploidy rates, which represent an intermediate outcome. Therefore, future research should examine whether embryo development directly impacts implantation or live birth rates, particularly after the transfer of euploid embryos. Second, the sample size was initially calculated based on a pilot study, which predominantly featured women aged ≥40 years old, which resulted in many aneuploid results. Third, time-lapse microscopy would provide better insights into the study of embryo development. Nevertheless, as mentioned earlier, time-lapse microscopy has been found to not be cost-effective and is not available at most centers, and we still have doubts about its benefits. In addition, the scope of this study was limited to embryos that reached the day-5 blastocyst stage; it excluded those that underwent slower development and formed blastocysts on day 6. Existing research generally indicates that day-6 embryos are less likely to be euploid and demonstrate reduced implantation rates [12,39].

This study has clinical implications for counseling infertile couples, particularly those where the female is of advanced age. PGT-A is widely used to improve live birth rates, but data are still conflicting if there are such benefits, as mentioned earlier. Previous reports of live births resulting from the transfer of mosaic embryos have raised concerns about the utility of PGT-A and the potential for misdiagnosis or spontaneous correction into euploid embryos [40]. With these limitations, there should be better ways to predict euploid embryos. Morphology and morphokinetics will also be beneficial in extraordinary situations when PGT-A is unfeasible, such as religious contexts, countries where PGT-A is prohibited [41], or due to financial constraints, and the findings of this study indicate that superior blastocyst expansion and TE grade can strongly indicate euploidy. Consequently, the strategic selection of embryos exhibiting favorable blastocyst and TE grades within this population could potentially enhance the chances of achieving conception earlier. In addition, this study showed that embryos with poor development and poor morphological grade were up to 73% aneuploid. These embryos should be prioritized last for embryo transfer if PGT-A is not performed.

This is the first study that evaluated the association between embryo development and chromosomal results using PGT-A in advanced-aged women. This study revealed a significant association between embryo development between the day-3 cleavage stage and the day-5 blastocyst stage and euploidy rates. Also, a meaningful relationship was found between blastocyst morphological grades and euploid rates, especially blastocyst expansion grade and TE grade. These findings indicate that the developmental morphological characteristics of blastocysts can provide valuable insights into their chromosomal status in advanced female age.

## 5. Conclusions

This study found a significant association between the development of embryos between the day-3 cleavage stage and the day-5 blastocyst stage and euploid status in advanced-aged women. Also, higher rates of euploidy were found in embryos with good blastocyst morphological grades. This insight requires further study of the other parameters studied to help develop ART success rates in advanced-aged women.

## Figures and Tables

**Table 1:** Classification of blastocyst morphological grade.

	Blastocyst Expansion Grade (Embryo Quality)	ICM*, TE** Grade
Good	4–6	AA, AB, BA
Moderate	4–6	BB
Poor	4–6 1–3	AC, BC, CC, CA, CB all

Abbreviations: ICM, inner cell mass; TE, trophectoderm.

**Table 2:** Baseline characteristics of infertile couples (n = 101).

Characteristic	Description
Age female (years) *	39.19 ± 2.61
≥35 to <38, n (%)	37 (36.63)
≥38 to <40, n (%)	24 (23.76)
≥40, n (%)	40 (39.60)
Age male (years) *	41.03 ± 5.07
BMI (kg/m^2^) *	22.89 ± 4.52
Nulliparity, n (%)	90 (89.11)
History of abortion, n (%)	27 (26.73)
Primary infertile, n (%)	69 (68.32)
Secondary infertile, n (%)	32 (31.68)
The primary cause of infertility, n (%)	
Male	15 (14.85)
Tubal	5 (4.95)
Ovulation	61 (60.40)
Endometriosis	4 (3.96)
Unexplained	16 (15.84)
Years of intended pregnancy (years) **	3 (2, 4)

* Data are expressed as the mean ± standard deviation. ** Data are expressed as median (interquartile ranges).

**Table 3:** Results from oocyte retrieval and ICSI and characteristics of embryos and PGT-A results.

	≥35 to <38 (n = 37)	≥38 to <40 (n = 24)	≥40 (n = 40)	Total	*p*-Value
Oocytes retrieved **	12 (7, 17)	9.5 (6, 15)	7.5 (4, 9)	8 (6, 15)	0.002
Mature oocytes (%) *	77.37 ± 15.41	83.69 ± 13.53	81.70 ± 18.85	80.58 ± 16.52	0.299
Fertilization rate (%) *	79.52 ± 15.60	79.56 ± 18.24	79.27 ± 21.37	79.43 ± 18.49	0.998
Cleavage rate (%)	100%	100%	100%	100%	1.000
Total day-3 cleavage **	8 (3, 12)	6 (3.5, 10)	4 (2, 6)	5 (3, 9)	0.007
Number of total blastocysts retrieved **	6 (2, 8)	3.5 (2, 8)	3 (2, 4)	3 (2, 7)	0.018
Blastocysts biopsied for PGT-A, n (%)	127 (39.08)	95 (29.23)	103 (31.69)	325 (100)	
Blastulation rate (%) *	69.02 ± 21.25	71.76 ± 22.89	72.44 ± 22.96	71.02 ± 22.16	0.785
Cleavage grade, n (%)					0.532
Good	105 (82.68)	79 (83.16)	80 (77.67)	264 (81.23)	
Poor	22 (17.32)	16 (16.84)	23 (22.33)	61 (18.77)	
Blastocyst morphological grade, n (%)					0.018
Good	6 (4.72)	0 (0)	2 (1.94)	8 (2.46)	
Moderate	42 (33.07)	38 (40.00)	24 (23.30)	104 (32.00)	
Poor	79 (62.20)	57 (60.00)	77 (74.76)	213 (65.54)	
Development, n (%)					0.008
Good	40 (31.50)	31 (32.63)	16 (15.53)	87 (26.77)	
Poor	87 (68.50)	64 (67.37)	87 (84.47)	238 (73.23)	
PGT-A results, n (%)					<0.001
Euploid	67 (52.76)	33 (34.74)	19 (18.45)	119 (36.62)	
Aneuploid	52 (40.94)	57 (60.00)	83 (80.58)	192 (59.07)	
Mosaic	8 (6.30)	5 (5.26)	1 (0.97)	14 (4.31)	

* Data are expressed as the mean ± standard deviation. ** Data are expressed as median (interquartile ranges).

**Table 4:** Association between embryo development and chromosomal results from PGT-A.

	Euploid, n (%)	Abnormal Chromosome, n (%)	*p*-Value
Good development, n (%) (n = 234)	48 (55.17)	39 (44.83)	<0.001
Poor development, n (%) (n = 91)	71 (29.83)	167 (70.17)	

**Table 5:** Univariate logistic regression analysis.

Variable	Value	Euploid Rate, n (%)	Abnormal Chromosome, n (%)	RR (95% CI)	*p*-Value
Female age	≥35 to <38	67 (52.76)	60 (47.24)	2.86 (1.85, 4.43)	0.001
	≥38 to <40	33 (34.74)	62 (65.26)	1.88 (1.15, 3.08)	0.011
	≥40	19 (18.45)	84 (81.55)	1	
Paternal age	≥35 to <38	24 (39.34)	37 (60.66)	1.13 (0.78, 1.64)	0.507
	≥38 to <40	24 (32.00)	51 (68.00)	0.92 (0.63, 1.36)	0.684
	≥40	60 (34.68)	113 (65.32)	1	
BMI (kg/m^2^)	<25	100 (38.31)	161 (61.69)	1	
	≥25	19 (29.69)	45 (70.31)	0.77 (0.52, 1.16)	0.220
History of abortion	Yes	32 (37.65)	53 (62.35)	1.04 (0.75, 1.43)	0.817
	No	87 (36.25)	153 (63.75)	1	
Cleavage cell	7, 8 cells	95 (35.98)	169 (64.02)	0.91 (0.64, 1.30)	0.618
	<7, >8 cells	24 (39.34)	37 (60.66)	1	
Blastocyst expansion grade	≥4	67 (53.60)	58 (46.40)	2.06 (1.55, 2.74)	<0.001
	<4	52 (26.00)	148 (74.00)	1	
ICM grade	A	8 (100.00)	0 (0)	-	
	B	105 (35.84)	188 (64.16)	1.43 (0.70, 2.91)	0.320
	C	6 (25.00)	18 (75.00)	1	
TE grade	A	8 (100.00)	0 (0)	-	
	B	97 (38.65)	154 (61.35)	1.82 (1.12, 2.98)	0.017
	C	14 (21.21)	52 (78.79)	1	
Blastocyst morphological grade	Good	8 (100.00)	0 (0)	-	
	Moderate	53 (50.96)	51 (49.04)	1.87 (1.40, 2.50)	< 0.001
	Poor	58 (27.23)	155 (72.77)	1	
Development	Good	48 (55.17)	39 (44.83)	1.85 (1.41, 2.43)	< 0.001
	Poor	71 (29.83)	167 (70.17)	1	

## Data Availability

Data are available upon reasonable request from corresponding author (matchuporn_m@yahoo.com).

## Supplementary Materials

The following supporting information can be downloaded at: https://www.mdpi.com/article/10.3390/jcm13020626/s1, Table S1: Aneuploid results from PGT-A. Table S2: Association of embryo development and chromosomal results from PGT-A in different age groups. Table S3: Multivariate logistic regression analysis.
