# Supplementary material for: The Association between Embryo Development and Chromosomal Results from PGT-A in Women of Advanced Age: A Prospective Cohort Study
Source: J Clin Med. 2024 Jan 22;13(2):626. doi: 10.3390/jcm13020626 (PMC10816670; doi:10.3390/jcm13020626)
Supplement: Supplementary file 1 [file jcm-13-00626-s001.zip › jcm-2783080-supplementary.pdf]

Supplementary Table S1 Aneuploid results from PGT-A

PGT-A results, n (%)	≥35 to <38 (n = 127)	≥38 to <40 (n = 95)	≥40 (n = 103)	Total (n = 325)	p-value
**Monosomy**	20 (38.46)	14 (24.56)	12 (14.46)	46 (23.96)	0.017
**Trisomy**	9 (17.31)	12 (21.05)	13 (15.66)	34 (17.71)	
**Complex**	23 (44.23)	31 (54.39)	58 (69.88)	112 (58.33)	

Supplementary Table S2 Association of embryo development and chromosomal results from PGT-A in different age groups

	Euploid, n (%)	Abnormal chromosome, n (%)	p-value
**⩾35 to <38**			
**Good development**	27 (67.50)	13 (32.50)	**0.024**
**Poor development**	40 (45.98)	47 (54.02)	
**⩾38 to <40**			
**Good development**	15 (48.39)	16 (51.61)	**0.052**
**Poor development**	18 (28.12)	46 (71.88)	
**⩾40**			
**Good development**	6 (37.50)	10 (62.50)	**0.072**
**Poor development**	13 (14.94)	74 (85.06)	

Supplementary Table S3 Multivariate logistic regression analysis

	RR (95% CI)	p-value
**Female age**		
**≥35 to <38**	2.48 (1.61, 3.83)	**< 0.001**
**≥38 to <40**	1.67 (1.03, 2.70)	
**≥40**	1	
**Development**		
**Good**	1.02 (0.73, 1.43)	**0.913**
**Poor**	1	
